# Supplementary material for: Excessive Fructose Intake Impairs Baroreflex Sensitivity and Led to Elevated Blood Pressure in Rats
Source: Nutrients. 2019 Oct 25;11(11):2581. doi: 10.3390/nu11112581 (PMC6893539; doi:10.3390/nu11112581)
Supplement: Supplementary file 1 [file nutrients-11-02581-s001.pdf]

nutrients

MDPI

Supplemental Tables

Table S1. Body weight, diet, drink and urine volume in groups subjected with or without 10% fructose water in the WKY rats.

**Control rats**	**Body weight (g)**	**Diet (g)**	**Drink (ml)**		**Urine (ml)**	
Day 0	224.00 ± 12.39	17.83 ± 0.76	34.50 ± 0.50		13.33 ± 2.44	
Day 1	224.33 ± 11.50	10.50 ± 1.64	33.33 ± 1.14		14.17 ± 1.64	
Day 2	226.50 ± 12.38	15.50 ± 0.65	35.50 ± 2.18		12.67 ± 1.45	
Day 3	230.17 ± 13.28	12.83 ± 0.50	34.67 ± 1.92		13.33 ± 1.40	
Day 4	230.83 ± 12.92	13.66 ± 1.33	34.33 ± 2.18		13.33 ± 1.99	
Day 5	233.00 ± 12.08	13.66 ± 2.13	36.00 ± 2.64		13.33 ± 1.40	
Day 6	235.17 ± 11.19	12.33 ± 0.70	34.83 ± 1.90		11.67 ± 1.47	
Day 7	235.17 ± 11.99	12.33 ± 1.48	35.33 ± 2.06		13.33 ± 1.33	
**Fructose-fed rats**	**Body weight (g)**	**Diet (g)**	**Drink (ml)**		**Urine (ml)**	
Day 0	224.50 ± 0.49	17.83 ± 0.60	29.17 ± 2.34		12.50 ± 1.45	
Day 1	226.67 ± 0.55	14.67 ± 0.98	49.83 ± 3.08	*	30.00 ± 2.46	*
Day 2	224.50 ± 1.02	14.17 ± 1.01	71.80 ± 4.46	*	49.67 ± 5.49	*
Day 3	225.83 ± 0.74	12.00 ± 0.93	62.50 ± 7.12	*	34.67 ± 5.85	*
Day 4	229.00 ± 1.09	12.00 ± 1.43	59.83 ± 5.46	*	36.17 ± 4.61	*
Day 5	231.00 ± 1.03	11.50 ± 0.88	58.00 ± 4.69	*	26.33 ± 3.88	*
Day 6	231.67 ± 0.66	11.17 ± 0.47	62.83 ± 3.82	*	33.33 ± 3.10	*
Day 7	230.17 ± 0.83	10.40 ± 0.87	66.83 ± 2.40	*	34.00 ± 2.58	*

Values are expressed as mean ± SEM. *$p < 0.05$ versus the control group.

Table S2. Triglycerides, HDL, LDL and total cholesterol levels in the serum in fructose-fed rats

	Triglycerides (mg/dL)			HDL (mg/dL)		LDL (mg/dL)		Total cholesterol (mg/dL)	
Day0	94.50	± 10.63		69.67	± 4.92	48.00	± 7.04	117.67	± 11.78
Day1	167.80	± 5.26	*	53.17	± 3.07	39.00	± 2.04	94.25	± 6.19
Day2	212.60	± 10.13	*	58.67	± 3.48	36.00	± 2.55	94.67	± 5.61
Day3	223.00	± 39.3	*	72.00	± 1.43	42.83	± 3.02	114.83	± 4.02
Day4	208.20	± 26.9	*	69.33	± 1.49	47.33	± 2.38	116.67	± 3.35
Day5	112.60	± 8.93		74.50	± 2.89	47.00	± 2.62	121.50	± 5.2
Day6	135.33	± 8.81		65.67	± 1.05	45.67	± 0.33	112.67	± 0.66
Day7	182.00	± 34.87	*	55.00	± 2.12	41.60	± 3.07	96.60	± 2.29

High-density lipoprotein (HDL) cholesterol, low-density lipoprotein (LDL) cholesterol. Values are expressed as mean ± SEM. *$p < 0.05$ versus the Day 0.

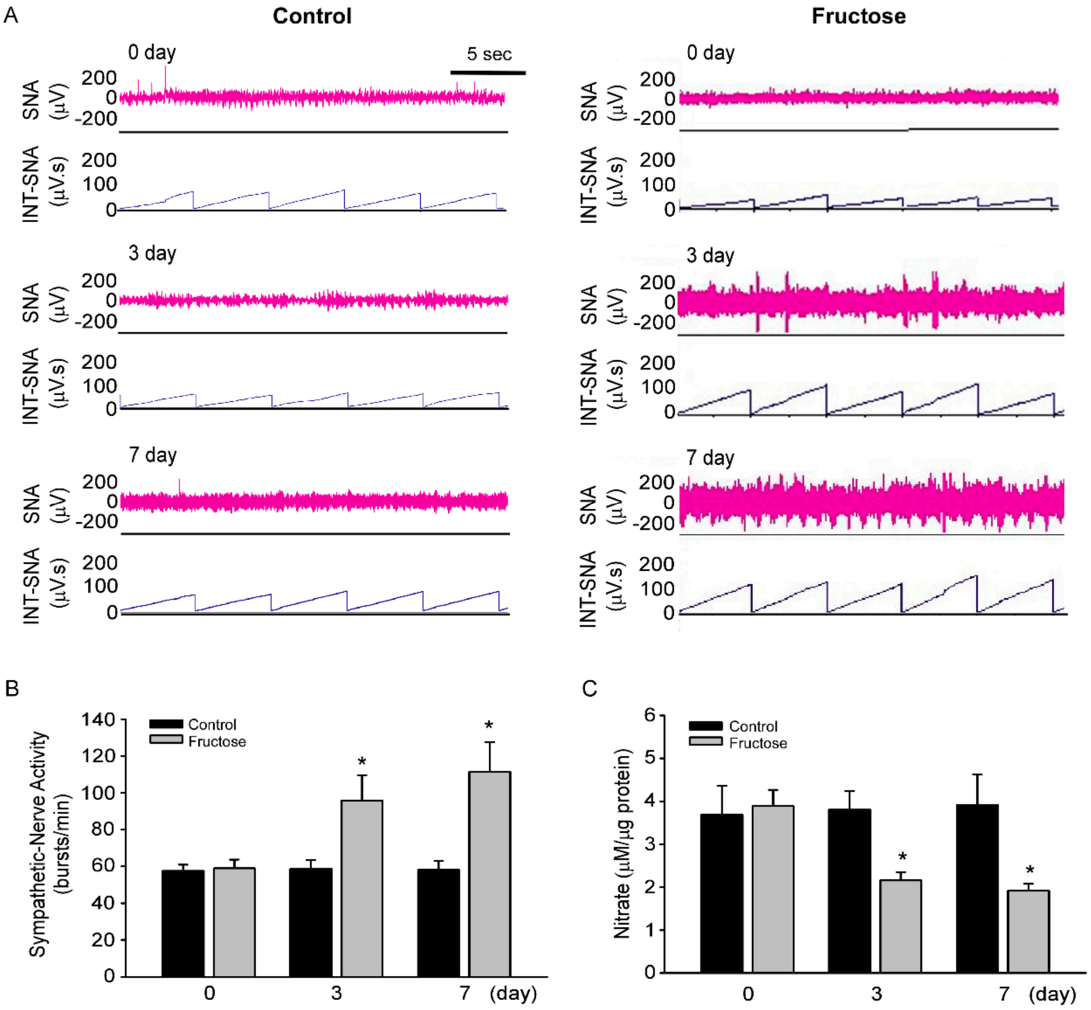

Supplemental Figure S1. Intake of 10% fructose water for 1 week increases SNA and decreases NO concentrations in the NTS. (A) Representative traces show the baseline renal SNA in fructose-fed rats throughout the 7 days of experiment. The time scale is one integrated value per 5 s. (B) Renal SNA in fructose-fed rats was measured on days 0, 3, and 7. (C) NO concentrations in the NTS of fructose-fed rats were determined throughout the 7-day research. The data are presented as the mean ± SEM (n = 5–7). *$p < 0.05$ versus the control group.

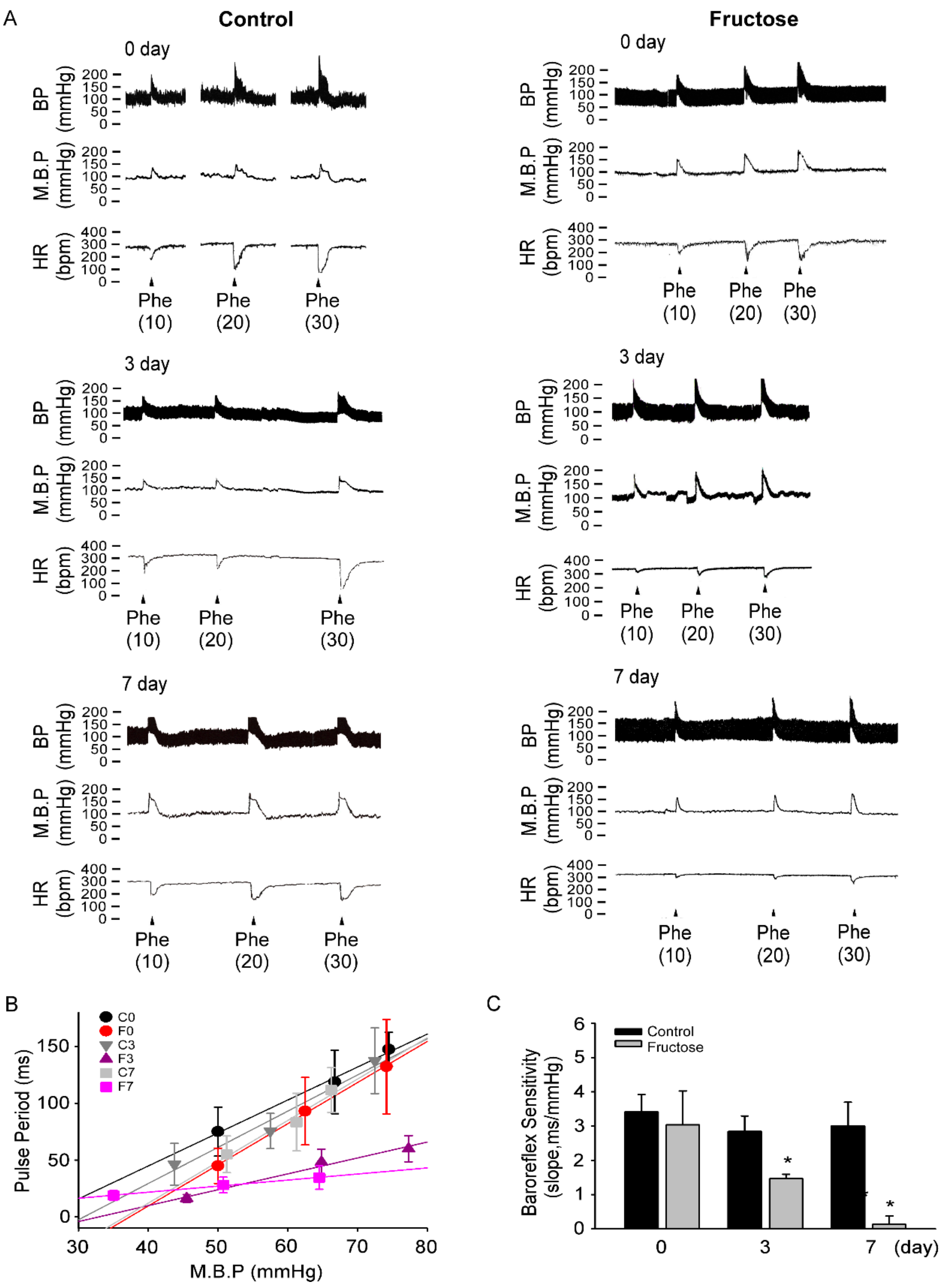

Supplemental Figure S2. Intake of 10% fructose water for 1 week impairs baroreflex response sensitivity. (A) Representative traces show baroreflex responses after intravenous injection of phenylephrine (Phe: 10, 20, 30 µg/kg) in fructose-fed rats on days 0, 3, and 7 of the experiment. (B) The points and vertical bars represent increases in the pulse period of the peak bradycardic response in response to the suppressive effects of different doses of phenylephrine. The lines connecting the points were obtained by linear regression analysis, which yielded the slopes of each group. (C) Effects of the NTS on baroreflex responses (slope) in fructose-fed rats on days 0, 3, and 7 of the experiment. The data are presented as the mean ± SEM (n = 4–6). *$p < 0.05$ versus the control group.
